# Unique presentation of superficial siderosis of the central nervous system following pituitary tumor surgery: a case report and literature review

**DOI:** 10.3389/fnimg.2026.1751864

**Published:** 2026-02-26

**Authors:** Tengyao Liang, Jiangqin Song, Fengzhu Zhao, Weifang Zhu, Hui Wei

**Affiliations:** 1 Tianmen Hospital Affiliated to Wuhan University of Science and Technology, Tianmen, Hubei, China; 2 The First People’s Hospital of Tianmen City in Hubei Province, Tianmen, Hubei, China; 3 Hubei Province Key Laboratory of Occupational Hazard Identification and Control, Wuhan University of Science and Technology, Wuhan, Hubei, China; 4 Department of Medical Laboratory, The First People’s Hospital of Tianmen City (Tianmen Hospital Affiliated to Wuhan University of Science and Technology), Tianmen, Hubei, China; 5 Department of Neurology, The First People’s Hospital of Tianmen City (Tianmen Hospital Affiliated to Wuhan University of Science and Technology), Tianmen, Hubei, China; 6 Department of Gastroenterology, The First People’s Hospital of Tianmen City (Tianmen Hospital Affiliated to Wuhan University of Science and Technology), Tianmen, Hubei, China; 7 Department of Cardiology, The First People’s Hospital of Tianmen City (Tianmen Hospital Affiliated to Wuhan University of Science and Technology), Tianmen, Hubei, China

**Keywords:** case report, central nervous system, cerebellar ataxia, pituitary tumor surgery, sensorineural hearing loss, superficial siderosis

## Abstract

Superficial Siderosis of the Central Nervous System is an infrequent neurological disorder resulting from hemosiderin deposition due to chronic and recurrent subarachnoid hemorrhage, leading to significant neurological impairments including sensorineural hearing loss, cerebellar ataxia, and pyramidal signs. This case report presents a 50-year-old male patient with a history of pituitary tumor surgery, manifesting progressive neurological symptoms over 2 years, thereby highlighting the potential long-term complications associated with SSCNS. The atypical clinical presentation, coupled with a surgical background, underscores the diagnostic challenges faced by clinicians, who may misattribute symptoms to more common neurological conditions. Advanced imaging modalities, particularly susceptibility-weighted imaging (SWI), have proven essential in enhancing the diagnostic accuracy for SSCNS, revealing characteristic patterns of iron deposition that are often subtle and can lead to delayed recognition. This case not only contributes to the existing literature by documenting a rare presentation of SSCNS but also emphasizes the necessity for increased awareness and vigilance among healthcare providers regarding this condition’s complex manifestations. The findings advocate for interdisciplinary collaboration between neurologists and radiologists to improve recognition and management strategies, ultimately leading to better patient outcomes. Despite the rarity and variability of SSCNS, which complicates the establishment of standardized treatment protocols, this case highlights the critical need for continued research into its underlying mechanisms and therapy efficacy, particularly in patients with previous neurological interventions. Enhanced educational initiatives may be pivotal in addressing the diagnostic challenges associated with this debilitating condition.

## Introduction

Superficial siderosis of the central nervous system (SSCNS) is an uncommon neurological disorder caused by chronic or recurrent bleeding into the subarachnoid space, leading to persistent exposure of the leptomeninges and subpial tissues to hemoglobin breakdown products and subsequent hemosiderin deposition on the surface of the brain, cranial nerves, and spinal cord (Lobo et al., 2022). Clinically, SSCNS is a progressive and potentially disabling condition that can markedly compromise quality of life and functional independence (Kharytaniuk et al., 2022). Although initially described by Hamill in 1908, systematic clinicopathological characterization and recognition of its typical clinical course and etiologic spectrum were established in the seminal work by Fearnley, Stephens, and Rudge (Katoh and Watanabe, 2023; Fearnley et al., 1995). More recently, a mechanistic framework has been refined, emphasizing iron-mediated neurotoxicity (including oxidative injury and secondary neuronal/glial damage) driven by ongoing or intermittent subarachnoid hemorrhage, thereby explaining the insidious progression and the importance of identifying and eliminating the bleeding source (Wilson et al., 2017).

The classical clinical phenotype predominantly comprises progressive sensorineural hearing loss, cerebellar ataxia, and pyramidal tract signs (often with additional myelopathic features) (Fearnley et al., 1995), which may mimic more common neurodegenerative or cerebrovascular disorders and contribute to underrecognition. Magnetic resonance imaging (MRI), particularly blood-sensitive sequences (e.g., T2*-weighted GRE or SWI), is central to diagnosis by demonstrating a characteristic hypointense rim along the pial surfaces and/or cranial nerves consistent with hemosiderin deposition; however, subtle or overlooked imaging findings can delay diagnosis (Wilson et al., 2017). Importantly, contemporary management paradigms increasingly stress a “source-directed” strategy—actively searching for persistent bleeding sources such as dural defects and considering definitive repair when feasible—given emerging evidence that targeted dural repair may reduce ongoing subarachnoid bleeding and potentially alter disease trajectory.

The case of a 50-year-old male patient with a history of pituitary tumor surgery presents a unique instance of SSCNS, underscoring the importance of considering this diagnosis in patients exhibiting chronic neurological symptoms following subarachnoid hemorrhage. The progressive nature of this patient’s condition over two years emphasizes the potential long-term complications associated with SSCNS, including irreversible neurological damage. This case not only contributes to the existing literature by highlighting a rare presentation of SSCNS in a patient with a specific surgical background but also reinforces the necessity for heightened awareness among clinicians regarding the condition’s atypical presentations and the implications for treatment and management strategies (McMahon et al., 2022).

## Case report

A 50-year-old man was admitted to the neurology department of our hospital in August 2020 with a 2-year history of dizziness and progressive unsteadiness while walking. He reported that the symptoms began after treatment for a pituitary adenoma and were accompanied by bilateral hearing decline and gradual cognitive complaints, predominantly affecting recent memory. He denied true spinning vertigo, nausea, vomiting, diplopia, dysphagia, choking on liquids, limb weakness, aphasia, seizures, or other focal neurological symptoms. He had sought medical attention at multiple institutions without meaningful improvement (details of prior medications were unavailable) and was therefore referred to our hospital for further evaluation. His medical history was notable for a pituitary adenoma diagnosed in 2016, treated with Gamma Knife radiosurgery, followed by craniotomy with tumor resection in June 2018. No interval MRI surveillance was obtained between 2016 and 2018 (between Gamma Knife treatment and craniotomy), mainly because of suboptimal follow-up compliance. He denied a history of hypertension, diabetes mellitus, stroke, coronary artery disease, head trauma, or toxic/drug exposures. There was no relevant family history and no known drug allergies.

Upon physical examination, the patient’s blood pressure was recorded at 124/82 mmHg, and no significant abnormalities were noted in the general medical examination. He was fully conscious and cooperative, though there was mild cognitive impairment indicated by a Mini-Mental State Examination (MMSE) score of 22. His speech was fluent, and both pupils were equal and reactive to light, measuring 2.5 mm. Ocular movements were normal, with no nystagmus observed. The nasolabial folds were symmetrical, tongue protrusion was midline, and bilateral hearing loss was evident. Muscle strength and tone in the limbs were normal, as were deep tendon reflexes. The finger-to-nose test was normal, but a positive Romberg test was noted. Both superficial and deep sensory functions were intact, and there were no pathological reflexes or signs of meningeal irritation. Bedside assessment revealed no spontaneous or gaze-evoked nystagmus. Orthostatic vital signs (supine-to-standing blood pressure measurement) did not meet criteria for orthostatic hypotension, and a supine roll test was negative for positional vertigo. Formal vestibular function testing (e.g., vHIT/HIMP or caloric testing) was not performed.

**Figure 1:**
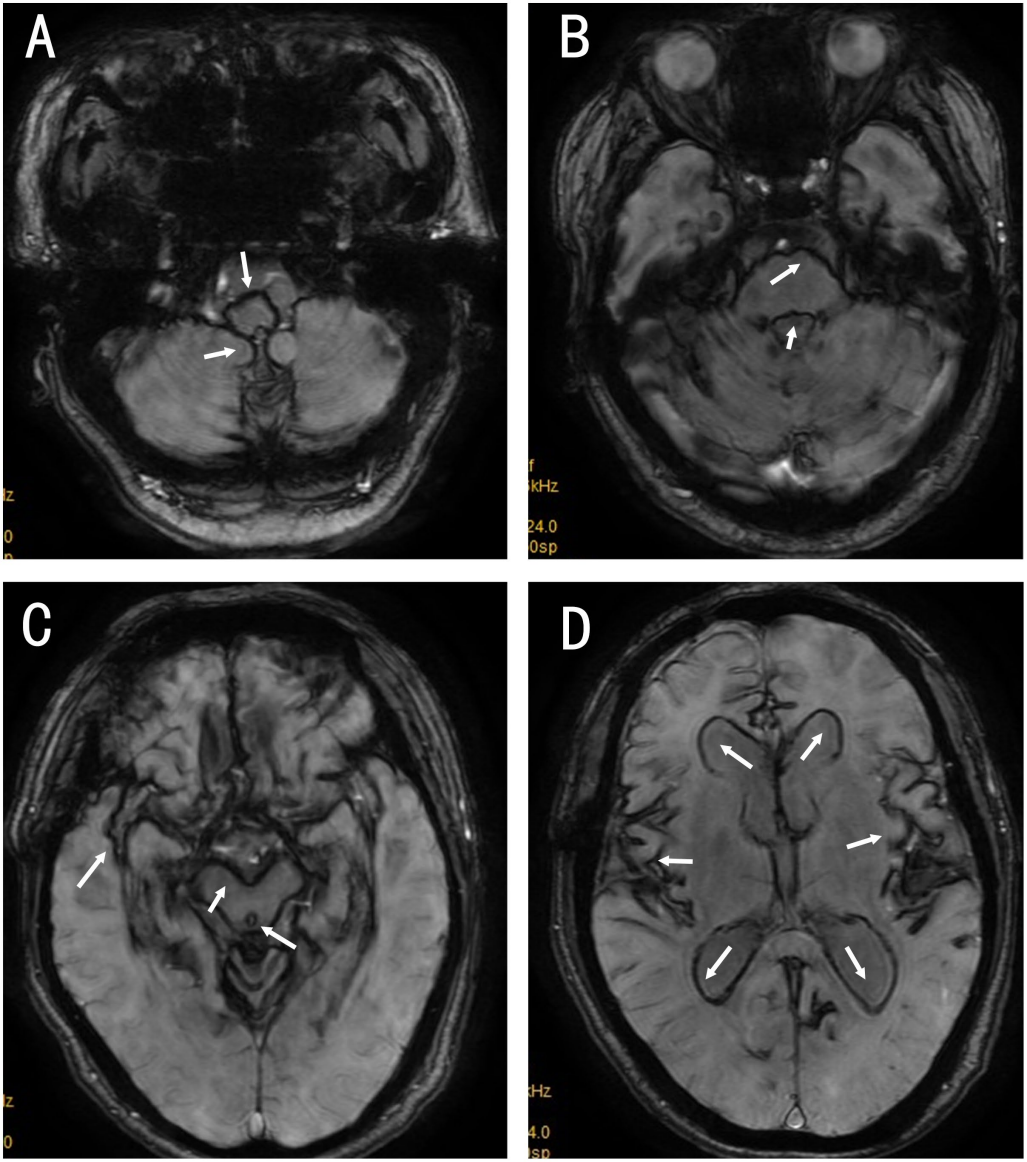
Non-contrast MRI **(A–D)**. T2-weighted imaging (T2WI) shows linear hypointensity along the pial and periventricular surfaces, predominantly infratentorial. **(A)** Linear hypointensity around the medulla oblongata (black arrow) and over the cerebellar surface (white arrow(s)). **(B)** Linear hypointensity around the pons and along the medial temporal lobe (white arrow(s)), with bilateral temporal lobe white-matter hyperintensities. **(C)** Linear hypointensity around the midbrain (white arrow(s)). **(D)** Linear hypointensity around the Sylvian fissure (white arrow(s)), with enlargement of the lateral ventricles and cerebral atrophy.

**Figure 2:**
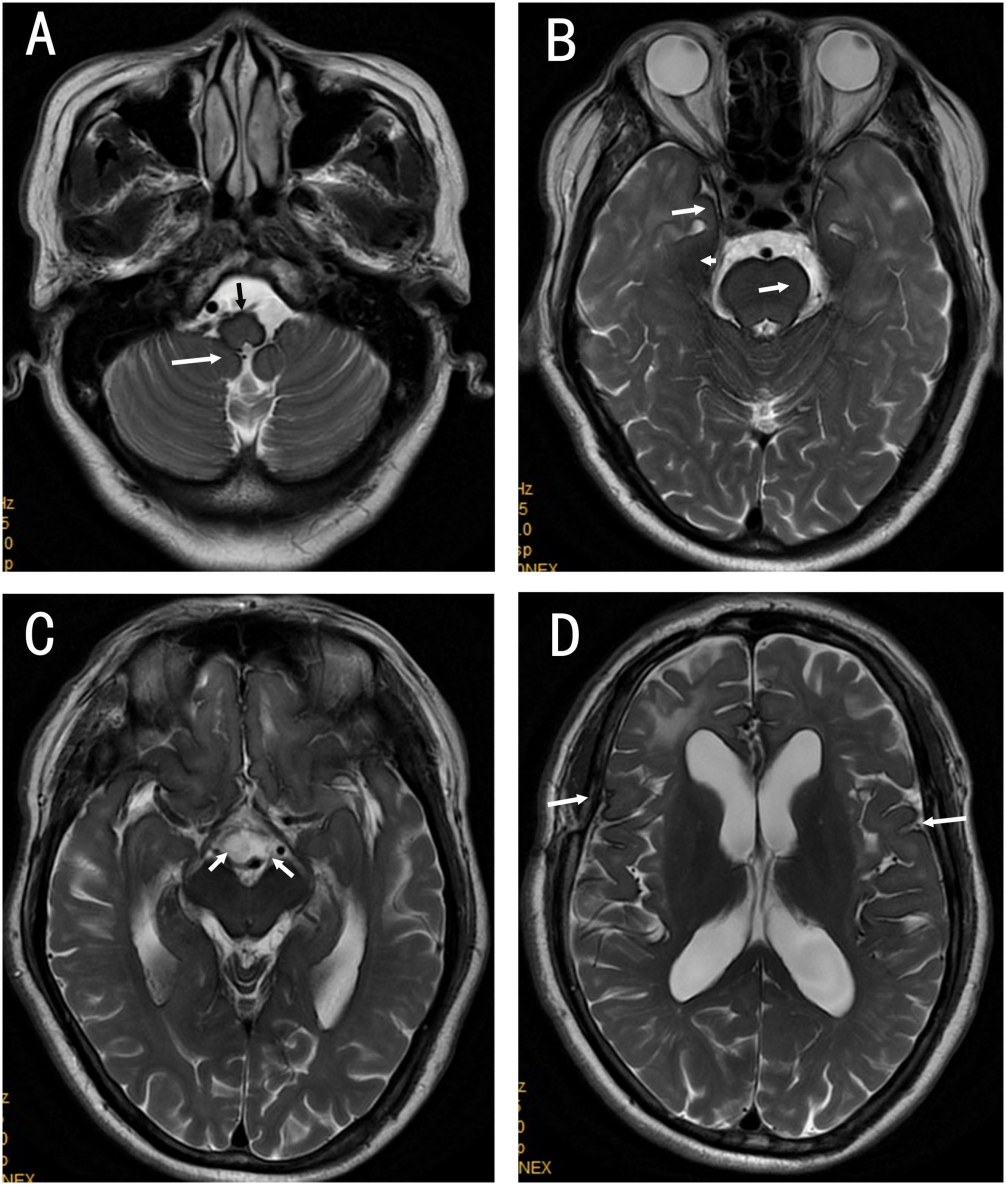
SWI **(A–D)** shows iron deposition along the cortical and periventricular surfaces. **(A)** Linear hypointensity around the medulla oblongata and over the cerebellar surface (white arrow(s)). **(B)** Linear hypointensity around the pons and the fourth ventricle (white arrow(s)). **(C)** Linear hypointensity along the temporal lobe surface, around the midbrain, and within the cerebral aqueduct (white arrow(s)). **(D)** Linear hypointensity around the lateral ventricles and the Sylvian fissures (white arrow(s)).

Laboratory tests revealed normal ranges for complete blood count, liver and kidney function, myocardial enzyme profile, coagulation function, D-dimer, homocysteine, and lipid profile. Tests for hepatitis B, hepatitis C, syphilis, and HIV were negative. Thyroid function tests indicated elevated thyroid-stimulating hormone (TSH) at 4.470 mIU/L (reference 0.4–4.0 mIU/L) and decreased free thyroxine (fT4) at 11.92 pmol/L (reference 12–22 pmol/L). Blood glucose indices showed an elevated hemoglobin A1c of 6.90% (diabetes diagnostic threshold ≥6.5%) and fasting plasma glucose of 6.33 mmol/L (ADA reference: normal <5.6 mmol/L, prediabetes 5.6–6.9 mmol/L, diabetes ≥7.0 mmol/L; WHO reference: normal <6.1 mmol/L, impaired fasting glucose 6.1–6.9 mmol/L, diabetes ≥7.0 mmol/L). Audiometry (Aug 4, 2020) showed bilateral high-frequency sloping sensorineural hearing loss. The pure-tone average (PTA; 0.5/1/2 kHz) was 36 dB HL in the right ear and 43 dB HL in the left ear (air conduction), with corresponding bone-conduction PTAs of 31 dB HL (right) and 38 dB HL (left). The air–bone gap was <10 dB HL bilaterally, supporting a sensorineural pattern. Based on PTA, hearing impairment was mild on the right and moderate on the left, with marked deterioration at higher frequencies (approximately 2–8 kHz) where thresholds reached the moderately severe to severe range. At the 2-year follow-up, the patient reported near-complete loss of functional hearing bilaterally, with inability to communicate by spoken voice. A cranial MRI scan revealed hypointensity in brain tissue on T2-weighted imaging, linear low signal on the brain surface, ventricular enlargement, white matter lesions, and brain atrophy (see Figures 1A–D). A neck and head computed tomography angiography (CTA) showed no evidence of vascular narrowing, malformations, or aneurysms. Suspected iron deposition was noted on susceptibility-weighted imaging (SWI), showing diffuse low signals on the surface of the pia mater, spinal meninges, and ventricular surfaces. Based on the SWI findings, a diagnosis of SSCNS was established (see Figures 2A–D).

The patient was treated with therapies aimed at improving cerebral circulation and nourishing the nervous system, which resulted in slight symptomatic improvement. But 2 years later, follow-up assessments indicated that the patient had experienced almost complete loss of hearing and significant cognitive decline, rendering him unable to care for himself.

## Discussion

In our patient, the florid infratentorial pattern of superficial siderosis on blood-sensitive MRI strongly supports a chronic or recurrent subarachnoid bleeding process rather than an isolated hemorrhagic event, consistent with established clinicopathological descriptions of SSCNS (Fearnley et al., 1995). From a mechanistic and practical perspective, current frameworks stress that ongoing iron-mediated injury drives clinical progression, making a source-directed workup essential to identify and eliminate the bleeding source whenever feasible (Wilson et al., 2017). This consideration is particularly relevant in the postoperative setting, where dural defects or occult spinal cerebrospinal fluid (CSF) leaks may serve as persistent bleeding foci; emerging evidence suggests that targeted detection and dural repair can reduce ongoing subarachnoid bleeding and may modify the disease trajectory (Lobo et al., 2022). Accordingly, beyond describing a rare association with a pituitary tumor history, this case underscores the need for systematic evaluation for a treatable bleeding source to potentially prevent further neurological decline.

A focused literature review was conducted in PubMed and regional sources using the keywords “superficial siderosis,” “pituitary adenoma,” “craniopharyngioma,” “dural defect,” “postoperative,” and “apoplexy,” restricting to human reports relevant to sellar or postoperative etiologies (Table 1). Cases due to non-surgical vascular lesions (aneurysm, arteriovenous malformation), major trauma, or systemic coagulopathies were excluded. Eight eligible cases were synthesized, including the present case (1998–2023). The cohort had a mean age of 56.5 years and was predominantly male. Two mechanisms predominated: tumor-associated hemorrhage (5/8; pituitary adenoma or craniopharyngioma) and postoperative dural defect/leak permitting chronic low-grade bleeding (3/8; one spinal dural tear and two postoperative sellar contexts). Progressive sensorineural hearing loss and gait ataxia were the leading manifestations; T2*-weighted GRE or SWI consistently revealed superficial siderosis along cerebellar and cortical surfaces. Management clustered into definitive tumor hemostasis/resection, targeted dural repair or CSF pathway reconstruction, and adjunct medical therapy (e.g., dopamine agonists for prolactinoma), with outcomes markedly better when the bleeding source was identified and controlled; in contrast, non-definitive care was associated with progression, including one postoperative death and persistent disability.

**Table 1:** Literature review of superficial siderosis of the central nervous system.

Reference	Year	Age/sex	Predisposing conditions	Presentation	Diagnosis	Etiology	Therapy/duration	Outcome
Sakamoto et al. (2004)	2004	37/F	Remote transsphenoidal subtotal resection of a pituitary adenoma (19 years earlier)	Progressive bilateral sensorineural hearing loss; cerebellar ataxia	Superficial siderosis of the central nervous system (SS)	Long-term, surgery-related recurrent microbleeding in the sellar region causing chronic subarachnoid hemorrhage	No iron chelation; conservative follow-up (duration not specified)	Hearing loss and ataxia continued to progress
Steinberg et al. (2013)	2013	43/M	Giant invasive prolactinoma with pituitary apoplexy	Headache; visual dysfunction; progressive hearing loss	Apoplexy-associated SS	Hemorrhagic pituitary macroadenoma leading to chronic/recurrent subarachnoid hemorrhage	Not reported	Not reported
Tosaka et al. (2015)	2015	69/M	Papillary craniopharyngioma of the third ventricle	Headache; gait instability; blurred vision; progressive hearing loss	Papillary craniopharyngioma with SS	Intratumoral hemorrhage leaking into the CSF circulation, resulting in a chronic hemorrhagic burden	Microsurgical tumor resection	Further postoperative decline in hearing
Oh et al. (2018)	2018	50/M	Papillary craniopharyngioma of the third ventricle	Intermittent vertigo and headache	Papillary craniopharyngioma with SS	Preoperative intratumoral hemorrhage with subarachnoid dissemination	Tumor resection	Postoperative death
Mapaga and Martinez (2020)	2020	58/M	Suspected pituitary macroadenoma with hyperprolactinemia	Three months of headache; no hearing loss at initial visit	Pituitary macroadenoma–related CNS SS	Presumed recurrent microbleeding from the adenoma; no alternative bleeding source identified	Cabergoline; MRI surveillance every 6 months	Clinically stable; adenoma decreased in size; superficial siderosis persisted
Matsuoka et al. (2022)	2022	75/M	Upper thoracic spinal dural defect (identified source of bleeding)	Progressive gait ataxia and hearing loss	SS due to dural defect	Dural tear causing chronic hemorrhagic CSF leakage	Surgical repair of the dural defect; symptoms stabilized postoperatively	No further progression after repair
Hashikata et al. (2023)	2023	70/M	Large nonfunctioning pituitary adenoma; SS present preoperatively	Headache; postoperative cerebral vasospasm and cerebral infarction	SS with postoperative vasospasm/infarction	Pituitary adenoma–related subarachnoid hemorrhage leading to SS; intraoperative hematoma entering the subarachnoid space may have increased hemorrhagic load	Endoscopic endonasal resection; CSF shunt for communicating hydrocephalus; antiepileptic therapy	Persistent impaired consciousness; poor functional recovery
Present case	2020	50/M	Prior pituitary adenoma interventions: Gamma Knife in 2016 and craniotomy for tumor resection in June 2018; symptom onset after surgery	Two-year history of dizziness and unsteady gait without true vertigo; bilateral sensorineural hearing loss; memory decline (MMSE 22); positive Romberg; no focal deficits	Superficial siderosis of the central nervous system	Presumed chronic/recurrent subarachnoid hemorrhage related to prior pituitary surgery; bleeding source not identified	Therapies to improve cerebral circulation and neurotrophic support; slight symptomatic improvement; duration not specified	At 2-year follow-up: near-complete hearing loss and marked cognitive decline with loss of independent self-care

SS, superficial siderosis; GRE/T2*, gradient-echo T2* sequence; SWI, susceptibility-weighted imaging; CSF, cerebrospinal fluid. Descriptions adhere to the reported items, standardize terminology, and state causal chains only where supported by the source; missing details are labeled “Not reported”.

Our patient fits a postoperative pathway: prior pituitary adenoma treatment (Gamma Knife radiosurgery in 2016 followed by craniotomy and tumor resection in 2018), craniospinal SWI evidence of diffuse siderosis, negative vascular imaging, and a chronic, low-grade course culminating in near-total functional deafness and cognitive decline. In this context, prior Gamma Knife radiosurgery and subsequent open surgery could be considered potential contributing background factors, as postoperative changes at the sellar region (including tissue remodeling and dural scarring) may, in principle, facilitate occult CSF-space bleeding or dural vulnerability, although a direct causal relationship cannot be established from the available data. Furthermore, the absence of interval MRI surveillance between 2016 and 2018 (mainly due to suboptimal follow-up compliance) may have limited early detection of progressive hemosiderin deposition and delayed etiologic evaluation. Taken together, these features are compatible with an occult dural or tumor-bed source that was not definitively localized or repaired. For similar cases, a source-first strategy is indicated—craniospinal SWI with targeted (dynamic) CT myelography/cisternography to localize small dural defects or CSF leaks, followed by watertight dural repair—before considering hearing rehabilitation.

In contrast to other studies, our case highlights a unique presentation following pituitary tumor Surgery, demonstrating the role of primary CNS tumors as potential bleeding sources—an aspect less frequently documented in the literature.

Imaging techniques, particularly susceptibility-weighted imaging (SWI), have revolutionized the diagnostic approach, allowing for more accurate identification of iron deposition patterns in SSCNS. While the existing literature outlines symptoms common to SSCNS patients, the variability in clinical presentation underscores the need for heightened awareness among clinicians. This case not only contributes to the understanding of SSCNS but also emphasizes the criticality of etiological exploration for effective management and highlights a significant gap in current literature regarding the long-term outcomes and treatment efficacy in similar cases. To frame the differential diagnosis, Table 2 summarizes MRI discriminators by distribution, T2/SWI morphology, ancillary sequences, and clinical context.

**Table 2:** SSCNS: key differential diagnoses on MRI.

Dimension	SSCNS	CAA-related cortical superficial siderosis (cSS)	Acute/subacute subarachnoid hemorrhage (SAH)	Cavernous malformation/microbleeds	Calcification	Melanin-related leptomeningeal disease	Cortical vein thrombosis
Typical location	Cerebellar folia, brainstem, CPA cisterns; may involve cranial nerves and spinal leptomeninges; infratentorial predominance, often symmetric.	Cortical convexities, occipito-parietal tendency; infratentorial uncommon; often coexists with lobar CMBs.	Basal cisterns or regional sulci by source; migrates with time.	Intraparenchymal clustered lesions; often adjacent to DVA.	Deep nuclei, choroid plexus, pineal region patterns.	Diffuse or patchy leptomeningeal involvement; pan-neuroaxis possible.	Along cortical venous trajectories.
T2*/SWI morphology	Continuous linear/laminar hypointensity hugging the surface; best seen with 3D-SWI/GRE; cranial nerves or spinal dura may be involved.	Convexity-limited linear or patchy hypointensity; SWI more sensitive than GRE-T2* for disseminated cSS.	Transient, discontinuous low signal that fades over time.	Round/ovoid blooming foci with a “popcorn” core; thin hemosiderin rim.	Marked low signal but typically non-leptomeningeal; confirm with phase or CT.	Low signal may be present but is nonspecific; prioritize T1 and enhancement.	Linear hypointensity along one or more cortical veins; intraluminal thrombus blooming; SWI highly sensitive.
Other sequences/contrast	None or mild enhancement; non-specific T1. Recommend brain + whole-spine SWI/3D-FLAIR/spine MRI; selective CT-myelography or DSM to localise a dural tear when suspected.	Distribution confined to convexities; often with white-matter markers (e.g., CSO-PVS, WMH-MS). Use SWI when available.	CT: acute hyperdensity; FLAIR sulcal hyperintensity; clear temporal evolution per guidelines.	Mixed T1/T2 core; possible thin rim enhancement.	CT first-line; d-SWI/p-SWI and phase images improve discrimination between calcium and blood products.	Unenhanced T1 hyperintensity plus diffuse leptomeningeal enhancement; FLAIR hyperintensity possible.	Confirm filling defect on MRV/CTV; venous infarct/hemorrhage may coexist.
Clinical context	Progressive sensorineural hearing loss and ataxia; chronic low-grade bleeding from spinal dural defect is common.	Older adults; TFNE; CAA background.	Thunderclap headache, meningeal signs; acute onset.	Seizures or focal deficits; familial forms exist.	Often incidental; non-specific symptoms	Associated melanocytic tumors or neurocutaneous syndromes.	Headache with focal deficits; prothrombotic states, infection, pregnancy/postpartum.

The diagnosis of SSCNS presents significant challenges, primarily due to its rarity and the nonspecific nature of its clinical manifestations. The presence of progressive neurological symptoms following a history of subarachnoid hemorrhage should prompt clinicians to consider SSCNS in their differential diagnosis. Misdiagnosis remains a notable concern, as common symptoms such as ataxia and hearing loss can often be attributed to other neurological disorders, leading to delays in appropriate management. The utilization of advanced imaging modalities, particularly susceptibility-weighted imaging (SWI), has proven essential in identifying the characteristic iron deposits associated with SSCNS, thereby facilitating earlier and more accurate diagnoses. This case underscores the necessity for an interdisciplinary approach, involving collaboration among neurologists and radiologists, to enhance recognition and treatment strategies for SSCNS (Liao and Deng, 2024).

Moreover, understanding the underlying pathophysiological mechanisms of SSCNS is crucial for developing effective therapeutic interventions. Chronic exposure to blood breakdown products in the subarachnoid space, particularly following recurrent hemorrhages, leads to hemosiderin deposition, which is implicated in oxidative stress and neurodegeneration (Raveendran and Rajini, 2024). As evidenced by this case, the association between SSCNS and prior surgical interventions, such as the resection of tumors, highlights a previously underappreciated etiological factor that warrants further investigation. Future research should aim to elucidate the long-term outcomes and efficacy of treatment approaches targeting the oxidative pathways involved in SSCNS, as existing management remains largely symptomatic (Mariajoseph et al., 2023).

The unique presentation following pituitary tumor surgery exemplifies the complexities associated with identifying SSCNS, particularly in patients with prior neurological interventions. Clinicians must maintain a high index of suspicion for SSCNS when faced with progressive neurological deficits, especially in the context of subarachnoid hemorrhage. The utilization of advanced imaging techniques, such as susceptibility-weighted imaging (SWI), is paramount in enhancing diagnostic accuracy and expediting appropriate management. Furthermore, the interdisciplinary collaboration among neurologists and radiologists is essential for improving recognition and treatment strategies for SSCNS, ultimately leading to better patient outcomes.

## Conclusion

SSCNS results from hemosiderin deposition and iron-mediated neurotoxicity secondary to chronic or intermittent subarachnoid hemorrhage. The typical phenotype comprises progressive cerebellar ataxia, sensorineural hearing loss, and pyramidal signs; in at-risk populations, SSCNS should be prioritized in the differential diagnosis and evaluated promptly. Susceptibility-weighted imaging (SWI) is highly sensitive for superficial iron deposition and should serve as the first-line modality, complemented by whole-brain and whole-spine imaging with systematic assessment of dural integrity to localize low-grade, intermittent bleeding sources, including postoperative dural defects, tumor-related leakage, and vascular malformations. Management centers on etiologic control: early identification and definitive repair or resection of the bleeding source can attenuate disease progression and improve functional outcomes, whereas pharmacologic approaches—including iron chelation—have limited evidentiary support and are unlikely to reverse established neurodegeneration. Multidisciplinary care and structured long-term follow-up are recommended to enable early recognition, timely intervention, and optimization of long-term prognosis.

In summary, the rarity of SSCNS, coupled with its complex clinical presentation and diagnostic challenges, necessitates a thorough understanding and consideration in clinical practice. Documenting such cases is vital for enhancing awareness and improving diagnostic accuracy, ultimately leading to better patient outcomes and informing future research efforts to elucidate the underlying mechanisms and potential therapeutic approaches for this debilitating condition.

## Data Availability

The original contributions presented in the study are included in the article/supplementary material, further inquiries can be directed to the corresponding author.
